# Can open data increase younger generations’ trust in democratic institutions? A study in the European Union

**DOI:** 10.1371/journal.pone.0244994

**Published:** 2021-01-06

**Authors:** Nicolás Gonzálvez-Gallego, Laura Nieto-Torrejón

**Affiliations:** Faculty of Law and Business Administration, San Antonio Catholic University, Murcia, Spain; Universidade da Coruna, SPAIN

## Abstract

Scholars and policy makers are giving increasing attention to how young people are involved in politics and their confidence in the current democratic system. In a context of a global trust crisis in the European Union, this paper examines if open government data, a promising governance strategy, may help to boost Millennials’ and Generation Z trust in public institutions and satisfaction with public outcomes. First, results from our preliminary analysis challenge some popular beliefs by revealing that younger generations tend to trust in their institutions notably more than the rest of the European citizens. In addition, our findings show that open government data is a trust-enabler for Millennials and Generation Z, not only through a direct link between both, but also thanks to the mediator role of citizens’ satisfaction. Accordingly, public officers are encouraged to spread the implementation of open data strategies as a way to improve younger generations’ attachment to democratic institutions.

## Introduction

A series of global-scale events and phenomena, such as the Great Recession and climate change, are shifting the way younger generations address economic, social and cultural issues and how they engage in the political life, as they are increasingly indifferent towards politics [1, 2]. In the European Union (EU), Millennials find living in a democracy less relevant than adults do and they support alternative systems largely than the rest of the population [3], probably because they feel themselves excluded from the traditional political system [4]. Then, although democracy still gets majority support, this attitudinal change can be a warning signal of some unmet demands and expectations. On the contrary, there are some other authors that stated that support for democracy remains during last decades and that there is no clear evidence for ensuring that younger generations distrust in democracy more than young people at any other moment of time, especially in the case of consolidated democracies [5, 6].

Most of the previous works that studied these issues among younger generations and age groups were based on measures on citizens’ support to the current political system, in contrast with other alternatives to democracy. Despite of these relevant contributions, some academics are querying the validity of those metrics to study the underlying support for democracy [7] and they prefer to analyze actual trust in democratic institutions to evaluate skepticism towards democracy [6, 7]. Although there are several views on trust, we consider the following definition to approach general trust: ‘trust is an expectancy of positive, or nonnegative, outcomes that one can receive based on the expected action of another party in an interaction characterized by uncertainty’ [8]. Based on this general conception, we can approach social trust, defined as a firm belief in the competence of an entity to act as expected. This belief is not permanently attached to that entity, but rather it depends on how it behaves in a given time and context [9]. Some other authors are more focused on psychological aspects of trust, and they define social trust as the psychological state that accepts vulnerability in the context of social interaction, subject to social risk and interrelationship among peers [10]. In this paper, we address another type of trust: institutional trust.

Institutional trust is a cognitive assessment of the relationship between trustors (citizens) and the trustee (public institutions) based on the expected utility of the latter performing satisfactorily [11, 12]. There is a consensus on considering institutional trust as an accurate measure that assesses the relationship between citizens and institutions [13]. In recent years, European citizens’ trust in institutions has fallen, existing different factors that explain this phenomenon. In advanced industrial societies, although economics are relevant, prosperity is shifting citizens’ values towards post-material goods, such as self-expression and active political participation, making citizens more critic toward hierarchy and authority [14]. However, macroeconomics still matter and it is widely agreed that the sovereign debt crisis started in 2009, together with the Great Recession, have collapsed trust [15, 16]. To manage this situation, European institutions and countries have been increasingly relying on technocratic elites, drawing more attention to competence and problem-solving than to fairness and citizen engagement in decision-making, leading European citizens’ distrust in official views and populism rising [17, 18]. It means that, although the crisis of trust in the EU is rooted in a deterioration of its economy, this has unveiled political factors that are also responsible for growing distrust. The Great Recession was a global crisis and, in the absence of an effective international financial institution that effectively provides support globally, national governments still were the public risk managers. Even in a multi-level government system like the EU and the Eurozone, there were no supranational risk-bearing mechanisms [19], which resulted in citizens disappointed with the public response given by national and European institutions and, consequently, more unconfident with those institutions [20].

Public officers and scholars are giving increasing attention to trust downturn because of the relevance of this indicator. It is generally agreed that keeping a certain level of trust in institutions is required to guarantee the stability of the economic system and the legitimacy of the market economy [21]. Also, a systemic level of institutional trust is necessary to make the political system work as well as to protect democracy and keep democratic institutions legitimated [22, 23]. Besides, institutional trust is also relevant because of its correlation with social or generalized trust, a key aspect of a flourishing society [24]. Social interactions used to occur in small communities and quite limited groups of people, but societies are becoming more complex and globalized, where individuals frequently communicate with strangers. Then, it is increasingly difficult to build social trust on the background of previous social interactions. Hence, in this context, institutions play a critical role in setting social common rules that reduce the uncertainty of social interactions and improve trust among citizens [25, 26]. As stated by Spadaro, institutional trust needs to be considered when explaining changes in interpersonal trust [25]. The link between institutional and personal trust is, therefore, relevant since the former can boost the latter and that improvement in social trust has a positive impact, leading to better economic conditions [27], making people happier and improving their subjective wellbeing [28].

But is there any consistent evidence of a substantial fall of trust in institutions among European Millennials? With due care, and according to the literature, there is no clear decrease in Millennials’ confidence towards national and European institutions [6]. The absence of conclusive findings relies, at least partially, on the fact that age is not usually entered as a core variable when studying trust. It implies that a deeper insight is required. However, some previous studies entered the age of the respondents as a control variable when measuring trust in European countries, leading to mixing results. Some of them revealed that young people are more confident in national institutions than the rest of the population [29–31], while in some other cases youngsters tend to trust less than older people [32–34]. However, regarding European institutions, most of the studies showed that age has a negative effect on trust, revealing that the younger the respondent is the higher his or her confidence will be [35–37]. In this paper, we contribute to this debate by measuring Millennials’ and Generation Z trust as a construct that comprises several individual measures of trust in a set of political institutions.

According to this scenario, governments and other institutions are developing open government strategies based on the release of open data to improve transparency with an ultimate expectation: promoting trust [38, 39]. There is also a lack of empirical studies on how open government data (OGD) affects European Millennials and Generation Z individuals in particular. Nevertheless, there is some evidence that suggests that its impact could be greater than for other age groups. Political and civic engagement is higher among youngsters, for they are more aware of internet’s potential role in generating and accessing public content [40]. They are also more willing to use internet-based services, such as social media, to complete their information collection processes [41]. Specifically, their high exposure to internet, used on a constant basis, is a feature that strongly determines how Millennials behave as digital natives [42], something that can be extended to Generation Z.

It is worth mentioning that, beyond open data, there are other determinants of trust that European governments should consider. One of them is the provision of services and previous citizens’ experience with them. Since individuals tend to trust if they have been fairly treated in the past, a positive experience with public services is expected to have a positive effect on institutional trust [43]. Macroeconomics are relevant too, as European citizens assess economic performance and they build trust in institutions accordingly, based on that evaluation [44]. The way Europeans perceive those economic indicators, as well as other performance measures, also affects citizens’ trust in institutions [45]. Absence of corruption is also a determinant of trust, since any allocation of public goods on the basis of connection and money violates the principles of a democratic government. Indeed, when citizens perceive that corruption increases, trust in public institutions decreases [46]. Consequently, public officers must pay attention to these objective and subjective drivers of trust. Concerning OGD, they should explore if open data policies can boost the effect of those determinants, and how they interact among them in order to promote citizens’ level of trust. Also they should study if open data and transparency can counterbalance negative episodes or events that lead to a decrease of institutional trust, such as an economic downturn.

The European Union is implementing different actions to promote open data and youth engagement. Recently, in 2019, the European Parliament released the Directive on open data and the re-use of public sector information. It explicitly recognizes the importance of an open government data environment in order to promote accountability and transparency. In 2018, the Council developed the EU Youth Strategy, the framework for youth policy cooperation among member states for 2019–2027. The first goal of this strategy is connecting EU with youth, since “an increasing number of young people lack trust in the EU, encounter difficulties in understanding its principles, values, and functioning. Democratic deficits in EU processes have also been identified as one of the reasons for rising Euroscepticism among young people.”

Finally, trust is commonly studied together with citizen’s satisfaction, since they are related. As stated by Weber et al. [47]: ‘trust is a future-based belief that politicians will be able to generate expected outcomes, whereas satisfaction is a retrospective view of outcomes generated by the government’. OGD leads to better public services that help to address social problems and concerns, adding social value for citizens [48, 49], who feel more satisfied. Then, when satisfaction with public sector performance increases, a positive effect on institutional trust is observed [50]. Therefore, it is reasonable to think that a mediation effect of satisfaction between open data and trust may exist.

Consistently with this review, the aim of this paper is to run a cohort analysis, including Millennial and Generation Z, which allows us to explore if open data strategies have a positive effect on trust in political and public institutions and if this relationship is mediated by citizen’s satisfaction. Following the criterion from Pew Research Center, we consider 1996 as the cutoff point. Therefore, those born between 1981 and 1996 are considered as Millennials and anyone born from 1997 onward is part of Generation Z [51]. In terms of age, when data used in this study was collected, Millennials were between 21–35 years old, and individuals from Generation Z were 20 years old or younger.

## Material and methods

### Data

We analyze citizens’ trust in national and European institutions from data of the European Social Survey (ESS). It is a cross-national survey conducted across the European Union every two years. Data for ESS Round 8, the one used for this work, was collected from September 2016 to December 2017. The ESS is an academically-driven survey that aims to monitor public attitudes and values in Europe, as well as to improve and consolidate methods of cross-national survey measurement. In Round 8, people from 23 European countries have been surveyed. The survey involves strict random probability sampling, a minimum target response rate of 70% and rigorous translation protocols.

We measure OGD implementation through the Open Data Barometer (ODB). It is produced by the World Wide Web Foundation and aims to reveal the prevalence and impact of open data initiatives. Data for this research was collected from July 2015 to June 2016 (Fourth Round) and then processed from August to December 2016. To obtain the ODB score, we considered readiness, implementation and impact of open data in each country. A greater value of this indicator implies a higher degree of openness. The sub-indexes, components and the general ranking rely on different sources: government self-assessment, peer-reviewed expert surveys, detailed dataset assessments and secondary data. For Round 4, 115 countries were included.

The overlap of ODB and data from ESS resulted in a final sample size of 20,696, and a sub-sample made up of 7,403 individuals (1,299 Z-Generation and 6,104 Millennials) from 18 European countries: Austria, Belgium, Switzerland, Czech Republic, Germany, Estonia, Spain, Finland, France, United Kingdom, Hungary, Ireland, Italy, Lithuania, Netherlands, Portugal, Sweden and Slovenia. Table 1 shows the list of core variables and their correspondent descriptive statistics.

**Table 1:** Descriptive statistics of the model variables.

	Mean	Min.	Max.	S.D.	Range
Trust in Parliament (TRSTPRL)	4.771	0	10	2.384	0–10
Trust in European Parliament (TRSTEP)	4.890	0	10	2.354	0–10
Trust in legal system (TRSTLGL)	5.682	0	10	2.423	0–10
Trust in politicians (TRSTPLT)	3.765	0	10	2.345	0–10
Trust in political parties (TRSTPRT)	3.823	0	10	2.298	0–10
Satisfaction with life (STFLIFE)	7.452	0	10	1.772	0–10
Satisfaction with the economy (STFECO)	5.212	0	10	2.161	0–10
Satisfaction with the government (STFGOV)	4.481	0	10	2.253	0–10
Satisfaction with democracy (STFDEM)	5.390	0	10	2.337	0–10
Satisfaction with education (STFEDU)	6.013	0	10	2.165	0–10
Sastisfaction with health (STFHLTH)	5.918	0	10	2.316	0–10
OGD score	57.799	23.300	100.000	18.950	0–100

This shows the following descriptive statistics: mean, minimum, maximum, standard deviation (S.D.) and range concerning all the variables involved in the model. *Note about scales*: *Trust variables*: *0 = no trust at all*, *10 = complete trust; Satisfaction variables*: *0 = extremely dissatisfied*, *10 = extremely satisfied*. *OGD*: *0 = No efforts; 100 = Highest efforts*.

Regarding to the set of variables that measures the trust dimension, the lowest value is presented by trust in politicians. On the contrary, citizens’ trust in legal system is the best valued variable. In relation to variables that outline citizens’ satisfaction in institutions, the lowest mean is reached by satisfaction with the national government. In addition, this variable shows the highest coefficient of dispersion (0.503), which reveals a high heterogeneity in the answers of respondents in relation to the satisfaction with this institution. On the other hand, satisfaction with life is the variable with the best average score and the lowest coefficient of dispersion (0.238).

### Preliminary tests

**Fig 1:**
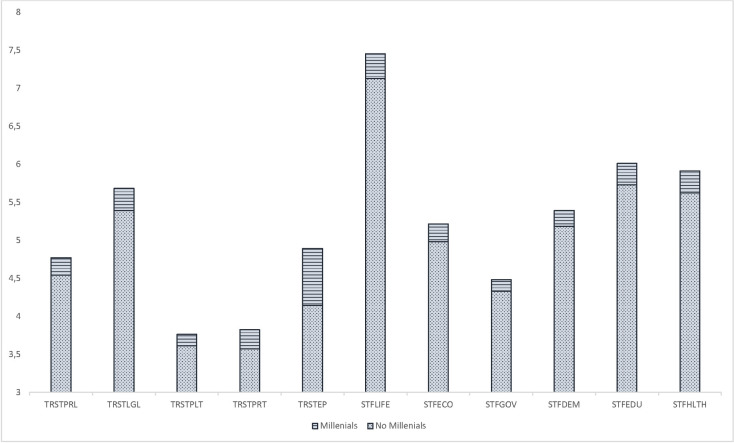
Comparison of mean values of institutional trust and satisfaction variables.

First, once data was collected, we split the whole sample into two smaller groups: one for Millennials and Generation Z (age<35) and another one for the rest of the population. In order to check if the level of trust is significantly different between these groups, we run a test for equal means. According to the results of this preliminary analysis (Table 2), significant differences (p<0.001) are shown for all the items. Additionally, we found that for all the indicators, individuals for younger generations feel more confident in public institutions than the rest of the sample (Fig 1). Then, we run the subsequent analysis exclusively referred to the Millennials and Generation Z sub-sample.

**Table 2:** Test for equal means.

Variable	Levene’s test (F-statistic)	t-test for equal means			
		t-statistic (d.f.)	Mean difference	Lower Coef. Int. 95%	Upper Coef. Int. 95%
TRSTPRL	58.181***	6.865*** (13726)	0.225	0.161	0.289
TRSTEP	82.781***	23.270*** (13530)	0.750	0.687	0.813
TRSTLGL	57.221***	8.924*** (13800)	0.298	0.232	0.363
TRSTPLT	13.434***	4.706*** (13313)	0.150	0.088	0.213
TRSTPRT	16.489***	7.977*** (13298)	0.250	0.188	0.311
STFLIFE	164.461***	12.540*** (14939)	0.314	0.265	0.363
STFECO	22.633***	7.756*** (13668)	0.230	0.172	0.288
STFGOV	60.572***	4.613*** (13788)	0.143	0.082	0.204
STFDEM	27.73***	6.309*** (13626)	0.202	0.139	0.265
STFEDU	22.709***	9.698*** (133601)	0.286	0.229	0.344
STFHLTH	74.109***	8.976*** (13728)	0.286	0.223	0.348

This test compares the difference between two independent groups with respect to the mean of an analysis variable. The standard error of the difference in means can be estimated using two formulas: (i) assuming equal population variances and (ii) assuming different population variance. Then, first we test the null hypothesis that the population variances of the two groups are equal using the Levene statistic. If the associated probability is less than 0.05, the hypothesis of equality of variances is rejected and they are assumed to be significantly different. Once the formula is determined, we check the bilateral significance of the t statistic. If p-value is greater than 0.05, the hypothesis of equality of means is rejected and we can confirm that there is a statistically significant difference and, if p-value is lower than 0.05, the hypothesis is accepted. *Note*:

***p < 0.*001*

; **p < 0.*05*.

### Structural equation modelling

To test the effect of open data on younger generations´ institutional trust, we developed a conceptual model to measure the direct effect of OGD on trust, as well as the indirect effect through satisfaction. To build this model we followed the structural equation modeling (SEM) methodology using IBM SPSS 24 AMOS software. The use of SEM allows us to hypothesize and confirm direct and indirect causal relationships among constructs, going beyond mere correlations, as well as to evaluate the whole model (goodness of fit) [52]. This methodology comprises two models: the measurement model and the structural one. The first one builds the constructs or latent variables through a confirmatory factor analysis, CFA, [53, 54], by extracting it from other variables and sharing the most variance with related variables [55]. For CFA, it has been used the Maximum Likelihood as factor estimation method and Varimax as the rotation procedure. The second stage of SEM is the development of the structural model. Throughout a path analysis, it enables to find the causal relationships among variables by creating a path diagram [56]. To perform the path analysis we followed four steps: (I) model specification, which consists of the definition of the hypothesized relationships; (II) model identification, which checks if the model is over, just or under-identified; (III) coefficients estimation when the model is just or over-identified; and (IV) model evaluation to assess overall goodness of fit.

Finally, we also capture the potential effect of micro variables (Table 3) on the relationships tested in the model. To do this, we performed a moderation (or multi-group) analysis for each path. Thus, we computed the differences in regression weights between groups defined by five moderating variables. As discussed, age shows mixed results and we included it in order to check if a cohort effect exists between Millennials and Generation Z. Besides, a higher level of education usually increases trust in public institutions [57]. Regarding gender, we found relevant the inclusion of this variable because, although it seems to lead to significant differences in trust when the whole population is studied, this gender gap could be demising among younger generations [58]. We also added the use of internet because although Millennials and Generation Z are digital native, we also want to explore if the effect of OGD, which is an internet-based tool, on trust is different between those connected to the internet on a daily basis and individuals who do not.

**Table 3:** Moderating variables. Descriptive statistics.

	Definition	Mean	Min.	Max.	S.D.	Range
**Gender**	Gender of the respondent	-	-	-	-	-
**Use of Internet**	How often the respondent use internet on different devices (computer, smartphones,…)	4.771	1	5	0.653	1–5
**Age**	Age of the respondent	26.103	15	35	5.811	15–35
**Education**	Highest level of education successfully completed by the respondent	4.172	1	7	2.742	1–7
**Ideology**	Where the respondent place himself on a scale	5.028	0	10	2.029	0–10

This table shows relevant descriptive statistics for the moderating variables. *Note about scales*: *Use of internet*: *1 = never*, *5 = every day; Education*: *1 = less than lower secondary*, *7 = higher tertiary education; Ideology*: *0 = Left*, *10 = Right*.

## Results

### Model specification

First, we developed a measurement model based on Gonzálvez-Gallego et al. [59]. Accordingly, there are eleven core variables (Table 1) and five moderators (Table 4). Then, we applied CFA in order to define the factors. Table 4 shows the two targeted factors get from the initial 11 items. Each factor groups those indicators with loadings above 0.3 [60]. The first factor gathers the five variables related to institutional trust, while the second one collects all the variables related to citizens’ satisfaction.

**Table 4:** Factor loadings for CFA.

	TRSTLGL	TRSTPLT	TRSTPRT	TRSTEP	TRSTPRL	STF LIFE	STF ECO	STF GOV	STF DEM	STFEDU	STF HLTH
**1**	**0.514**	**0.883**	**0.883**	**0.614**	**0.679**	0.088	0.322	0.433	0.384	0.240	0.251
**2**	0.446	0.315	0.287	0.326	0.454	**0.371**	**0.693**	**0.677**	**0.679**	**0.471**	**0.438**

This matrix shows the pattern of loadings on the factors, which allows to identify the two latent variables (in bold) of this model.

In order to confirm factors validity, we checked internal correlations between items and if they share a high proportion of variance [61]. Accordingly, Average Variance Extracted (AVE) was 0.5 and 0.4 for factor 1 and factor 2, respectively. Composite Reliability (CR) reached 0.8 for factor 1 and 0.7 for factor 2. AVE and CR values were above the required thresholds [62], so that we can confirm constructs validity.

Additionally, Cronbach’s alpha coefficient for factor 1 was 0.9 and for factor 2 was 0.8. In both cases, higher than the recommended value by Fornell and Bookstein [63] y Hair et al. [61]. Then factors internal reliability is confirmed.

### Model identification

In the second step we verify that the model is identified, which requires the following:

$$df\geq \frac{v(v+1)}{2}-p$$ (1)

where *df* are the degrees of freedom, *v* is the number of measured variables (endogenous and exogenous) and *p* is the number of parameters to be estimated. Given a total of 27 variables (13 endogenous and 14 exogenous) and having to estimate 29 parameters, the degrees of freedom result 349. Since the number of degrees of freedom is greater than zero, the model is over-identified.

### Model estimation

**Fig 2:**
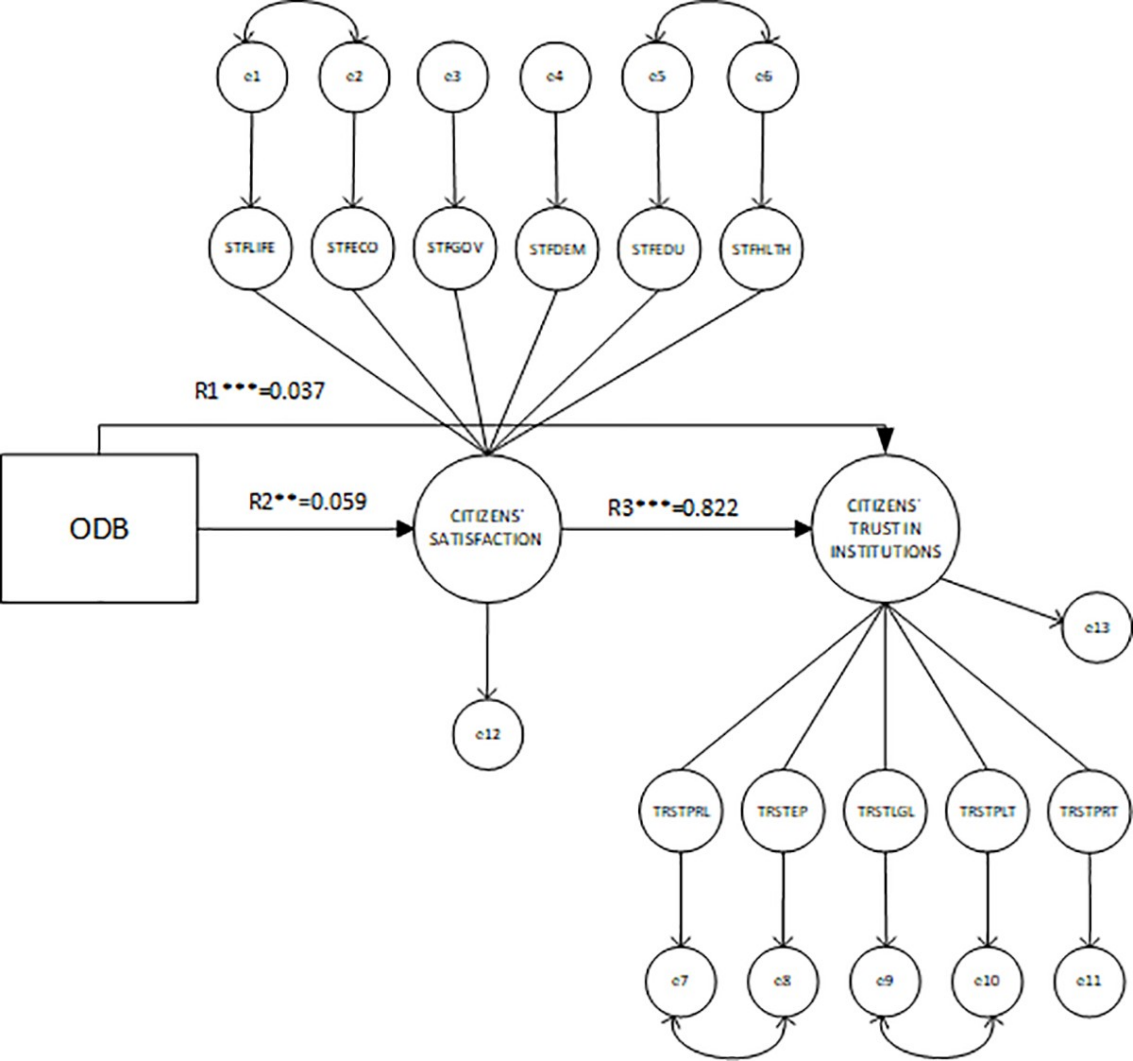
Measurement model. The rectangle depicts the implementation of OGD measured by the ODB score. The two largest circles are the latent variables for trust and satisfaction, two formative constructs built up from six and five indicators, respectively. Figures next to or above the arrows are the standardized regression weights, which are significant at a 0.001 level (***), except R_1_ which is significant at a level 0.05 (**). Residual (error) variables were added for every endogenous variable.

Fig 2 represents the structural model, it details how the latent constructs are determined from the observed variables and the standardized regression weights for the three proposed relationships: OGD→TRST (R_1_), OGD→STF (R_2_), and STF→TRST (R_3_); and for the independent variables.

Regression weights for *R*_*1*_, *R*_*2*,_ *and R*_*3*_ are positive and significant. This confirms both the direct impact of open data on institutional trust and the indirect effect between these two when the relationship is mediated by citizens’ satisfaction. The strongest link is R_3_ with a standardized regression weight of 0.822, while the weakest one is the R_2_, for which the standardized coefficient is 0.059. Because of this, we can conclude that the direct effect of open data on trust (0.037) is lower than the indirect effect when this relationship is mediated by satisfaction (0.048).

### Model evaluation

The overall evaluation determines to what extent the model adequately reproduces the existing relationships in the covariance matrix of data. Then, model fit determines the degree to which the proposed structural equation model fits the sample data.

There is no single statistical significance test that allows identifying a correct model given the sampled data; it is necessary to take into consideration different criteria to assess the model fit on the basis of multiple measures [64]. Thus, to validate the model specification, in this paper we use the following indicators: Normed Fit Index, NFI [65]; Tucker-Lewis Index, TLI [66]; Comparative Fit Index, CFI [66] and Goodness of Fit Index, GFI [67]. All the indexes meet the thresholds, so that the proposed structural model achieves a satisfactory goodness of fit (Table 5).

**Table 5:** Model fit indexes.

Index	NFI	TLI	CFI	GFI	AGFI	RMSEA
**Threshold**	>0.9	>0.9	>0.95	>0.9	>0.85	<0.08
**Model**	0.967	0.956	0.968	0.966	0.944	0.065

This table compares the values obtained for different indexes in our model with the values that are generally accepted by the literature.

### Moderating variables analysis

The criteria followed to split the sample into groups and the results obtained are shown in Table 6.

**Table 6:** Groups definitions.

Variable	Group Description	Name of group	Description	Sample size	R_1_		R_2_		R_3_	
					Z-Score	p-value	Z-Score	p-value	Z-Score	p-value
**Gender**	1	G1	Male	3,708	-0.685	N.S.	-2.124	0.05	-1.322	N.S.
	2	G2	Female	3,695						
**Age**	0–20 years	A1	Z-Generation	1,299	0.420	N.S.	-1.074	N.S.	0.811	N.S.
	20–35 years	A2	Millennials	6,104						
**Use of internet**	1–3	U1	From 1 (never) to 4 (most days use)	400	2.850	0.01	0.425	N.S.	0.203	N.S.
	4–5	U2	Daily use	7,001						
**Education**	1–5	E1	Less than advanced sub-degree	5,545	0.334	N.S.	2.976	0.01	3.172	0.01
	6–7	E2	Tertiary education	1,826						
**Ideology**	0–4	I1	Left	2,136	-1.174	N.S.	1.600	N.S.	0.535	N.S.
	5	-	Center*	3,068						
	6–10	I2	Right	2,199						

*Group description* column shows how we categorized the respondents in two different groups per moderating variable. For *Ideology*, the centered group (score 5) is out of the analysis. Also, p-values for critical ratios of the tested relationships are included (R_1_, R_2_ and R_3_) to determine the significance of the difference between groups. *Note*: *N*.*S*. *Not significant*.

The level of education is the single variable that significantly moderates the relationship between OGD and institutional trust, revealing that a higher educational level implies a greater confidence in institutions. The coefficient for respondents with tertiary education is higher (β_E2_ = 0.111) than for those less educated (β_E1_ = 0.044).

The relationship between OGD and satisfaction is moderated by gender and ideology. OGD has a weaker and negative effect on satisfaction for females (β_G2_ = -0.014) than for males (β_G1_ = 0.039). Concerning ideology, the size of the effect is, in absolute terms, higher for those that are self-positioned on the right (β_I2_ = -0.071) than for individuals who are self-positioned on the left (β_I1_ = 0.029). In addition, the former effect has a negative influence.

Finally, the relationship between satisfaction and trust is exclusively moderated by ideology. The influence of this variable is higher for people that is positioned on the right (β_I2_ = 0.913) than for leftists (β_I1_ = 0.821).

The only variables that do not show a moderating effect in any of the three relationships are internet use and age. The lack of moderating effect of the former variable could be explained by the fact that the 94.6% of respondents make an intensive use of internet. In terms of the age of the respondents, the absence of a moderating effect implies that there are no significant differences between Z-generation and Millennials.

## Discussion and conclusion

First, contrary to the popular belief, our results show that younger generations are more confident on public institutions, especially in European ones. This is a relevant contribution since most of previous studies evaluated this relationship by comparing their support to democracy versus other alternatives. In this paper, we measured trust, a consensual variable in the literature to evaluate the link between citizens and political institutions.

Second, for the sub-sample of Millennials and Generation Z, we confirm a direct effect of OGD on trust as well as an indirect impact through citizens’ satisfaction. This reveals that younger people value the efforts made by governments to be more transparent and they tend to trust more in public institutions when they release public and open data. Consequently, public officers should strengthen the implementation of transparency policies based on open data to continue promoting trust among these cohorts. It is worth noticing that the size of the indirect effect is higher that the direct one. It means that they feel more satisfied with public outcomes when institutional openness is higher. It could be because transparency and open data lead to a more efficient and better public management that derives a better experience for citizens and, as a consequence, they increase their confidence in institutions. Another possible explanation is that open data promotes accountability, and making control to government’s action make Millennials and Generation Z feel more satisfied with democracy, public services and the economy. As a result of this, their trust increases also.

Third, results from the moderation analysis reveal some significant contributions. Thus, the effect of open data on trust, when mediated by satisfaction, is higher for those self-positioned on the right, in contrast with the whole population [59]. For individuals from the analyzed cohorts with tertiary education, a higher direct effect is also observed. These findings suggest that conservatives derive more satisfaction from open data than their leftist peers do, and they transfer that level of satisfaction to institutional trust to a higher extent. Also, there is no cohort effect between Millennials and Generation Z on the relationships tested in our model.

Finally, it is necessary to highlight that, although all the hypothesized relationships have been confirmed, the size of the effects is still small. Our findings are aligned with previous studies that state that open government data, as an area of smart government, is still in its early stages of development [68]. Moreover, the release of open data by public institutions is not enough to generate benefits for citizens and other stakeholders [69]. There are also some organizational barriers that are limiting the adoption of open data policies, such as the increasing difficulty of managing an open government ecosystem [70]; public officers’ risk-averse attitude [71]; and the cost governments incur because of the adoption of those measures [72]. However, as found in our study it does not mean that openness effort does not generate a proportionate impact on society. Indeed, as a technology-based innovation, open data policies are being progressively adopted. Most of the European countries started to develop those strategies and tools during the last decade, so that it is soon to determine their effect on citizenship. Nonetheless, results are promising and we encourage public officers and academia to continue measuring how open data improves people’s trust, especially from the younger cohorts, in the long term on a regular basis.

Despite the relevance of our contribution, our study has different limitations and consequently, we suggest some recommendations for further research. First, we captured the effects in a certain moment of time but we can expect that, as open data tools are developed and increasingly used by citizens, the size of the effect will be higher. This means that, as more data is expected to be available in the future, longitudinal studies will contribute to shed light on this field. Second, as individuals grow up, the effects may differ. Then, monitoring these cohorts will provide valuable insight on the link between open data and trust. Third, we explored the effect of open data on institutional trust, but other relationships could be adressed, for instance, in order to study if open data boosts the relationship between institutional trust and generalized trust [73]. Fourth, this study is focused on the European Union; replicating similar experiments in other countries or regions will allow to make comparisons and explore different patterns that explain the links analyzed in this paper.
